# Identifying sinonasal inverted papilloma by machine learning: a systematic review and meta-analysis

**DOI:** 10.3389/fonc.2025.1628999

**Published:** 2025-08-26

**Authors:** Xianfei Qin, Jinping Shi, Xiangkun Zhao, Yu Zhang, Xueyan Liu, Li Wang

**Affiliations:** ^1^ The Second School of Clinical Medicine, Binzhou Medical University, Yantai, Shandong, China; ^2^ Liuzhou Traditional Chinese Medicine Hospital, Liuzhou, Guangxi, China; ^3^ Otorhinolaryngology Head and Neck Surgery, Yantai Yuhuangding Hospital, Yantai, Shandong, China

**Keywords:** machine learning, meta-analysis, radiomics, sinonasal inverted papilloma, systematic review

## Abstract

**Background:**

Sinonasal inverted papilloma (IP) is a benign tumor of the sinonasal mucosa, which may become malignant. Machine learning (ML) has been applied to improve the accuracy in the diagnosis of various diseases, but no studies have evaluated the performance of ML for IP diagnosis. This systematic review and meta-analysis aimed to explore the diagnostic performance of ML for IP.

**Methods:**

We systematically searched articles from PubMed, Cochrane, Embase, and Web of Science up to July 22, 2025. The quality assessment of diagnostic accuracy studies tool (QUADAS-2) was used to assess the risk of bias, and the bivariate mixed-effect model was used for meta-analysis.

**Results:**

17 studies involving 3321 participants were included. In the validation set, the sensitivity and specificity of ML constructed based on radiomics for identifying IP and malignant tumors were 0.84 (95%CI: 0.77-0.89) and 0.82 (95% CI: 0.74 ~ 0.88), respectively. The sensitivity and specificity of ML constructed based on radiomics and clinical features for identifying IP and malignant tumors were 0.85 (95%CI: 0.78-0.90) and 0.87 (95% CI: 0.80 ~ 0.92), respectively.

**Conclusion:**

Our study shows that ML has a favorable performance in the differential diagnosis of IP. More prospective studies are needed to validate and develop universal tools.

**Systemic Review Registration:**

https://www.crd.york.ac.uk/PROSPERO/view/CRD42023430417, identifier CRD42023430417.

## 1 Introduction

Sinonasal inverted papillomas (IPs) are benign but locally aggressive tumors that arise from the Schneiderian membrane in the sinus cavity with a high rate of recurrence and a tendency toward malignant transformation (1). The incidence of this tumor is 0.2 to 1.5/100,000 people per year with a higher incidence in men, and it affects individuals of all age groups (1). The clinical symptoms and imaging characteristics of IP closely resemble malignant tumors, often leading to diagnostic confusion. However, their prognosis and treatment strategies differ significantly (2). Therefore, accurate preoperative diagnosis of sinus tumors is essential for developing appropriate treatment strategies and assessing patient outcomes.

The diagnosis of IP can be made from clinical manifestations, imaging and pathological examination. However, the results are not satisfactory (1), especially in areas with underdeveloped medical resources. Radiomics, a field focusing on extracting image features quantitatively from standard medical imaging, has emerged as a valuable tool for improving diagnostic accuracy, prognosis assessment, and predictive capabilities in clinical decision support systems (3). Radiomics is widely used in the qualitative analysis, therapeutic effect evaluation and prognosis prediction of various tumors, and has developed rapidly in the field of tumor management (4).

Most studies have been conducted to distinguish IP from malignant tumors, while few studies focus on evaluating the diagnostic performance of machine learning methods. Therefore, we conducted this study to explore the diagnostic value of machine learning methods in IP diagnostics.

## 2 Materials and methods

### 2.1 Study registration

This study was conducted in accordance with reporting guidelines for systematic reviews and meta-analyses (PRISMA_2020) and prospectively registered on Prospero.

### 2.2 Eligibility criteria

Inclusion criteria were as follows:

1. The participants of this systematic review were individuals with nasal lesions;
2. The included studies were case-control studies, cohort studies and cross-sectional studies;
3. Studies has to construct a machine learning model for differential diagnosis of sinonasal inverted papilloma;
4. Some studies had difficulty achieving external validation, but they only provided K-fold cross-validation or internal validation of random sampling. Although it might overstate the accuracy of the model, its value was still fairly representative. Therefore, studies without external validation were also included in this systematic review and meta-analysis.
5. Studies that used the same dataset to construct different machine learning models were also included;
6. Studies written in English were included.

Exclusion criteria were as follows:

1. The research types were meta-analyses, reviews, guides, expert opinions, etc.;
2. Studies that only performed univariate analysis but did not construct a machine learning model were excluded;
3. The outcome indicators such as the Roc, c-statistic, c-index, sensitivity, specificity, accuracy, recovery rate, accuracy rate, confusion matrix, diagnosis four-cell table, F1 score, and calibration curve were missing to evaluate the accuracy of the machine learning models;
4. Studies with a sample size of less than 20 cases were excluded.

### 2.3 Data sources and search strategy

We searched the literature via PubMed, Cochrane, Embase, and Web of Science, up to July 22, 2025. The retrieval used subject-specific and free-text keywords, and the subject headings encompassed “machine learning” and “Nose Neoplasms”, with no restriction on the region. The detailed search strategy is shown in **Supplementary Table S1**.

### 2.4 Study selection

We used endnotes to filter the literature. The literature screening was completed by two investigators. After completion, they cross-checked their results to ensure accuracy. Disputes, if any, were resolved by a third investigator.

### 2.5 Data extraction

Prior to data extraction, we designed a standardized spreadsheet that included information such as title, first author, year of publication, country of author, type of study (case-control, cohort study (retrospective, prospective), nested cohort study, case-cohort study), patient origin (single center, multicenter, registry database), diagnostic criteria for inverted papilloma, reference subjects, number of inverted papilloma cases, total number of cases, the number of inverted papilloma cases in the training set, total number of cases in the training set, generation method of validation set [internal validation (random sampling, K-fold cross-validation, leave one method), external validation (prospective, multicenter)], overfitting method, number of cases of inverted papilloma in validation set, number of cases in validation set, variable screening/feature selection method, type of model used, and modeling variables (radiomics, clinical features).

The data extraction was completed by three independent investigators. After completion, they cross-checked their results to ensure accuracy. Dissents, if any, were resolved by a third investigator.

### 2.6 Risk of bias in studies

The risk of bias in the included studies was assessed using the diagnostic test evaluation tool QUADAS-2 (5). It assesses the risk of bias in data compilation and the clinical applicability of the original diagnostic test. QUADAS-2 is composed of 4 domains: patient selection, index test, reference standard, and flow and timing (5). Each domain contains several specific questions with three response options: “yes,” “No,” or “inconclusive,”. The responses correspond to a “low,” “high,” or “uncertain” risk of bias, respectively. If the answers to all the questions are “yes”, then the risk of bias is deemed to be low; if any of the questions receive “no”, then there is a possibility of bias, and the evaluator must determine the risk of bias according to the established guidelines. An “uncertain” risk means that the literature lacks detailed information to make a conclusive judgment. **Supplementary Figure S2** provides a comprehensive overview of the risk of bias assessment using the QUADAS-2 approach for each study included in the analysis.

### 2.7 Outcomes

Results Measures included sensitivity, specificity, positive likelihood ratio, negative likelihood ratio and diagnostic odds ratio. Sensitivity and specificity were extracted from the ROC curve and combined with the number of cases, a four-cell diagnostic table was made.

### 2.8 Synthesis methods

Data analysis was conducted by using Stata15.0 (StataCorp LLC, College Station, TX). A bivariate mixed-effect model was used to analyze the differential diagnosis of IP. The combined sensitivity, specificity, positive likelihood ratio, negative likelihood ratio, diagnostic odds ratio and 95% confidence interval (95% CI) of the effect size were calculated, and the area under the integrated receiver operating characteristic (SROC) curve was estimated. The Deek funnel plot was used to assess publication bias. P<0.05 indicated a significant difference between/among treatments.

## 3 Results

### 3.1 Study selection

We initially identified 2,111 studies, of which 193 were duplicates (143 duplicated articles were automatically detected by software and 50 duplicated articles were manually removed). After screening the title and abstract, 1,885 articles were further deleted, and the full texts of the remaining 33 articles were downloaded. Subsequently, we evaluated the full texts and discarded 16 articles for several reasons: three conference abstracts were published without peer review; in 10 studies, the control events were non-IP, and only image segmentation was performed; two studies did not construct a complete model; and one study investigated the accuracy of unresponsive machine learning. Finally, we included 17 studies for further analysis (6–22). The literature screening process is illustrated in **Supplementary Figure S1**.

### 3.2 Study characteristics

The 17 eligible studies were published between 2016 and 2024. Among the included studies, 13 studies focused on IP and malignancy (6–10, 13–15, 18, 19, 21–23); 3 studies investigated inverted papilloma and nasal polyps (11, 16, 17), and one study investigated inverted papilloma and sinusitis (12). Of the studies that identified IP and malignancy, eight studies constructed a machine learning model based on radiomics (7, 9, 10, 13–15, 18, 23), and seven studies constructed a machine learning model based on radiomics and clinical features (6–9, 13, 14). Studies on IP and nasal polyps constructed machine learning models using radiomics, whereas the studies on IP and sinusitis constructed machine learning models based on radiomics and clinical features. Six studies focused on automatic segmentation from images using deep learning methods (6, 11, 13, 14, 16, 18). **Table 1** lists the basic features of the included studies.

**Table 1:** Basic features of the included studies.

First author	Year	Country	Study type	Patient source	Number of sinonasal inverted papilloma cases	Total number of cases	Number of sinonasal inverted papilloma cases in training set	Total number of cases in training set	Generation of validation set	Number of sinonasal inverted papilloma cases in validation set	Total number of cases in validation set	Model type
Meng Qi (24)	2023	China	Case-control	Single center	135	209	90	140	Random sampling	45	69	Stepwise logistic regression, Decision curve
Marn Joon Park (7)	2023	Korea	Case-control	Single center	325	370	301	345				Logistic regression
Duo Zhang (8)	2023	China	Case-control	Single center	21	104	21	104				Logistic regression
Yang Yan (9)	2022	China	Case-control	Single center	144	236	94	157	Random sampling	50	79	Logistic regression
George S. Liu (10)	2022	America	Case-control	Multi-center	64	90	51	72	Random sampling	13	18	CNN
Xinyao Li (11)	2022	China	Case-control	Single center	52	136	45	114	Random sampling	7	22	CNN
Zengxiao Zhang (12)	2022	China	Case-control	Single center	267	540	200	400	Random sampling	67	140	logistic regression
Jinming Gu (13)	2022	China	Case-control	Single center	106	247	58	135	10- fold cross verification, Random sampling	25 23	58 54	logistic regression (LR), support vector machine (SVM), Decision tree (DT), and K-nearest neighbor (KNN)
Han Zhang (19)	2020	China	Case-control	Single center	113	197	80	138	Random sampling	33	59	logistic regression
Chong Hyun Suh (15)	2021	Korea	Case-control	Single center	41	62	41	62				Decision tree
TAO REN (16)	2021	China	Case-control	Single center	49	136	39	114	10- fold cross verification, Random sampling	8	22	Logistic regression
Benton Girdler (17)	2021	Korea	Case-control	Single center	100	297	100	297				CNN
Shu-cheng Bi (18)	2021	China	Case-control	Multi-center	126	244	102	192	Random sampling	24	52	Logistic regression
Lisong Zhang (19)	2020	China	Case-control	Single center	190	268	190	268				Logistic regression
S. Ramkumar (23)	2017	America	Case-control	Single center	22	46	16	33	Prospective validation	6	13	
RyosukeYui (21)	2023	Japan	Case-control	Single center	21	53	21	53	Fold cross verification			CNN
Yimin Ren (22)	2024	China	Case-control	Single center	37	86	37	86	fold cross verification			Logistic regression

### 3.3 Risk of bias in studies

In the included retrospective study, a diagnostic model based on machine learning was constructed, and the modeling variables were mostly radiomic characteristics. Therefore, no high risk of bias was found. The modeling variables in the included studies were radiomic features, identifying diseases according to machine learning rules. Therefore, knowledge of the gold standard does not create a risk of biased results. IP was diagnosed by pathological biopsy, so the risk of bias in all studies was low. There is an appropriate time interval between the IP evaluation test and the gold standard. Each patient receives the same gold standard. The evaluation results are shown in **Supplementary Figure S2**.

### 3.4 Meta-analysis

#### 3.4.1 IP vs. malignant tumors

##### 3.4.1.1 Training set

**Figure 1:**
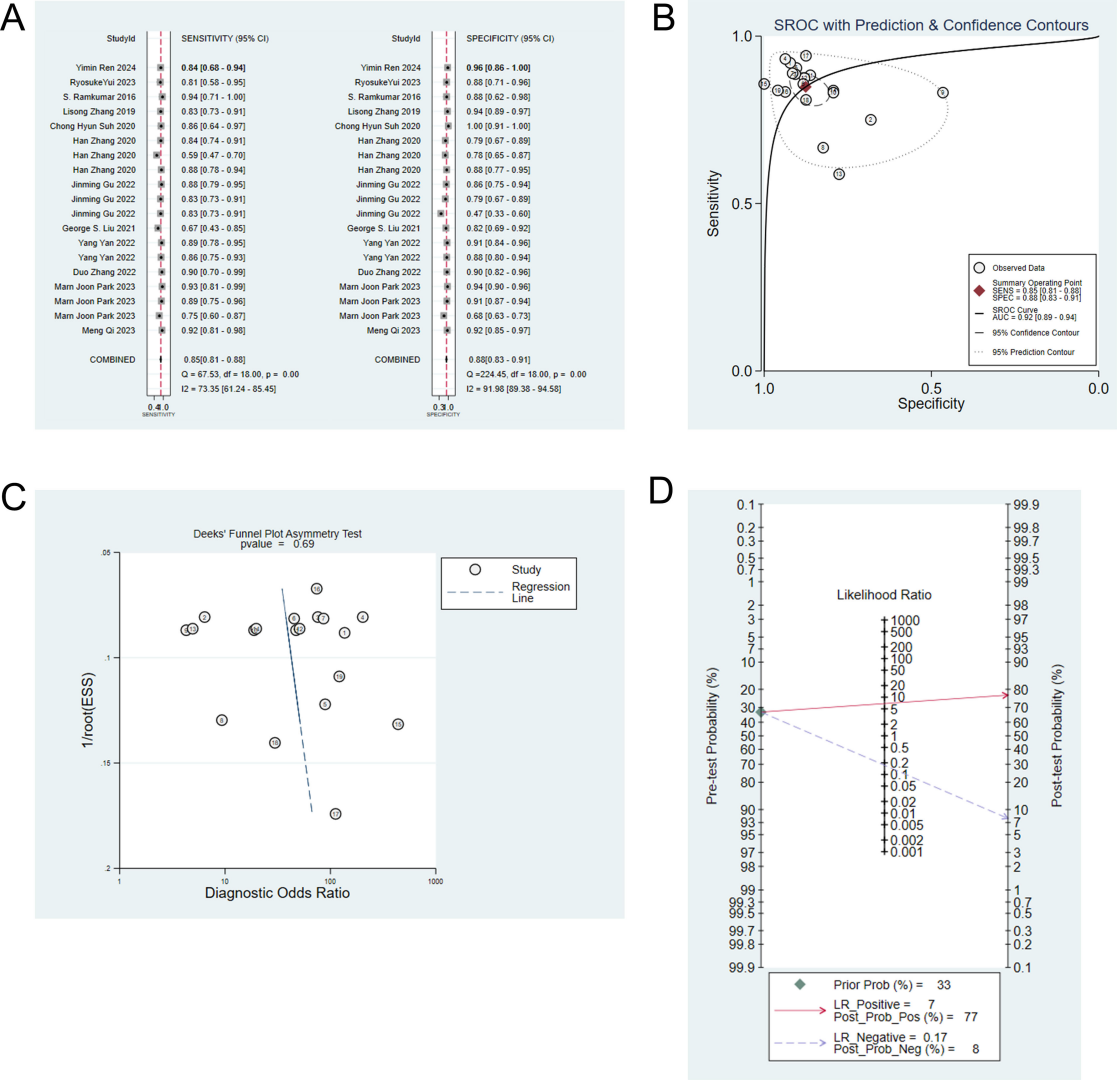
**(A)** Forest map of machine learning for identifying IP and nasal malignancies in the training set. **(B)** SROC of machine learning for identifying IP and nasal malignancy in the training set. **(C)** Funnel plot of machine learning for identifying IP and nasal malignancies in the training set. **(D)** Nomogram of machine learning for identifying IP and nasal malignancies in the training set.

Twelve studies (6–10, 13–15, 19, 21–23) employed machine learning to differentiate IP from malignant tumors. The pooled sensitivity, specificity, positive likelihood ratio, negative likelihood ratio, and diagnostic odds ratio of machine learning to differentiate IP from malignant tumors were 0.85 (95% CI: 0.81-0.88), 0.88 (95%CI: 0.83-0.91), 6.8 (95%CI: 4.7-9.8), 0.17 (95%CI: 0.13-0.23), and 40 (95%CI: 22-70), respectively (**Figures 1A, B**). Publication bias was not detected among these models in the training set (**Figure 1C**). The prevalence of IP in the included studies was 33%. Based on this prevalence, we established a prior probability of 33%. If the machine learning suggested IP, then the posterior probability of diagnosis of IP in positive patients was 77% and that of diagnosis of IP in negative patients was 8% (**Figure 1D**).

**Figure 2:**
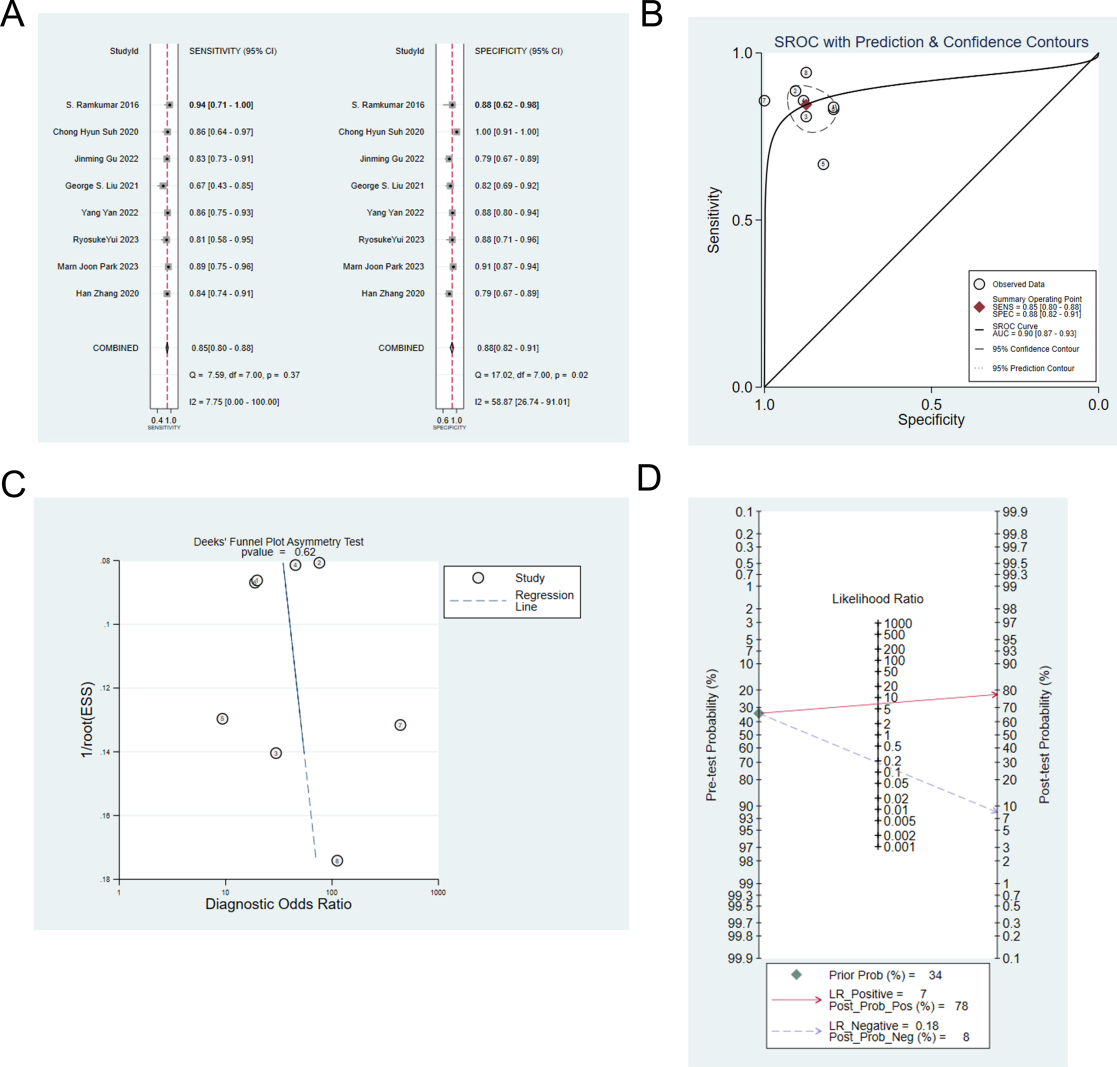
**(A)** Forest map of machine learning based on radiomic for identifying IP and nasal malignancies in the training set. **(B)** SROC of machine learning based on radiomic for identifying IP and nasal malignancy in the training set. **(C)** Funnel plot of machine learning based on radiomic for identifying IP and nasal malignancies in the training set. **(D)** Nomogram of machine learning based on radiomic for identifying IP and nasal malignancies in the training set.

Eight studies (7, 9, 10, 13–15, 21, 23) constructed machine learning models based on radiomic for the differential diagnosis of IP and malignant tumors. The analysis showed that the sensitivity, specificity, positive likelihood ratio, negative likelihood ratio and diagnostic odds ratio of the models for identifying IP and malignant tumors were 0.85 (95% CI: 0.80-0.88), 0.88 (95%CI: 0.82-0.91), 6.8 (95%CI: 4.7-9.8), 0.18 (95%CI: 0.13-0.24), 38 (95%CI: 21-68), respectively (**Figures 2A, B**). Publication bias was not identified among these models in the training set (**Figure 2C**), and the prevalence of IP in the included studies was approximately 34%. Based on this prevalence, we established a prior probability of 34%. If the machine learning suggested IP, then the posterior probability of diagnosis of IP in positive patients was 78% and that of diagnosis of IP in negative patients was 8% (**Figure 2D**).

**Figure 3:**
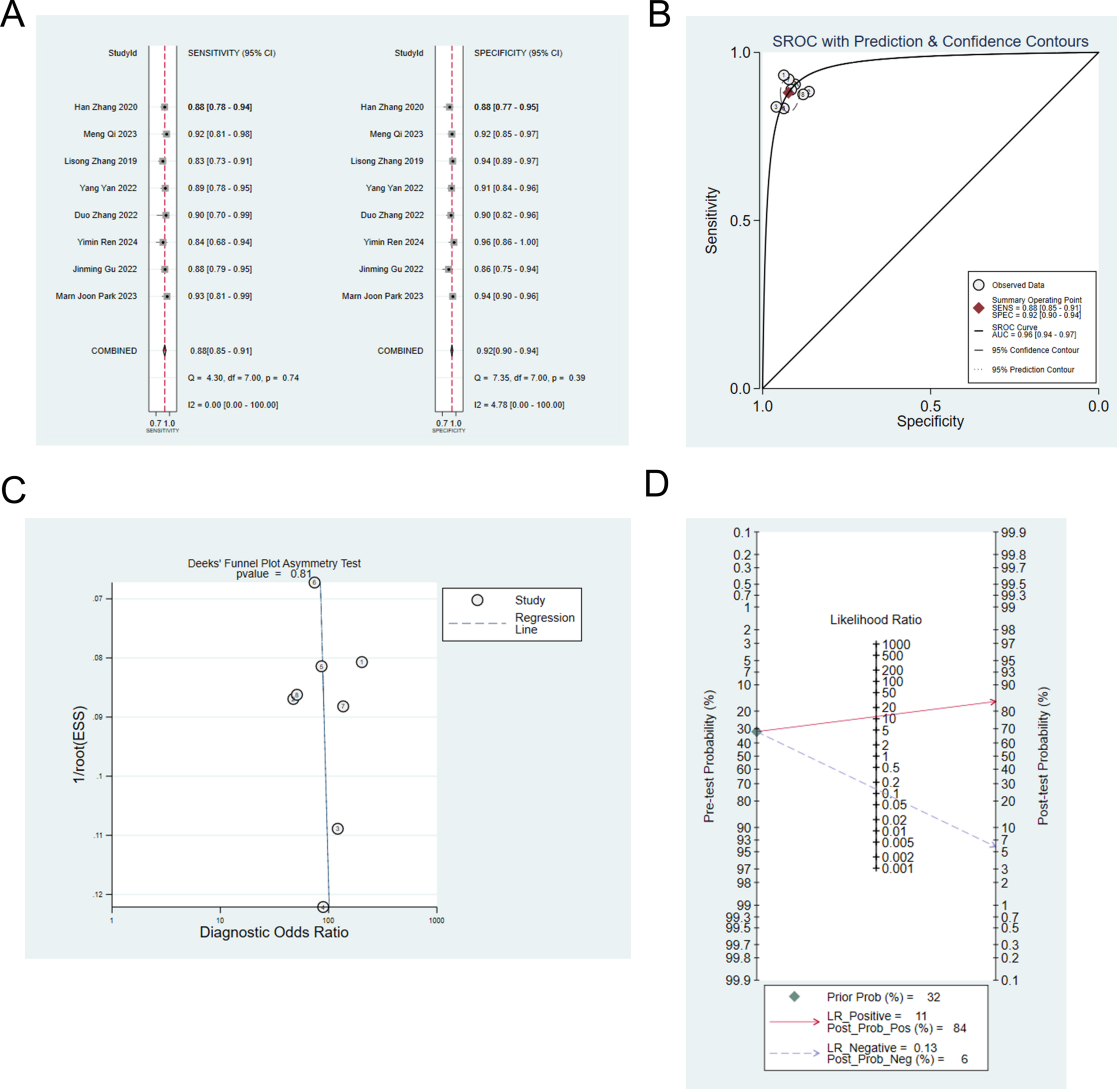
**(A)** Forest map of machine learning based on radiomic and clinical features for identifying IP and nasal malignancies in the training set. **(B)** SROC of machine learning based on radiomic and clinical features for identifying IP and nasal malignancy in the training set. **(C)** Funnel plot of machine learning based on radiomic and clinical features for identifying IP and nasal malignancies in the training set. **(D)** Nomogram of machine learning based on radiomic and clinical features for identifying IP and nasal malignancies in the training set.

Eight studies (6–9, 13, 14, 19, 22) constructed machine learning model based on radiomic and clinical features to differentiate IP from malignant tumors. The analysis showed that the sensitivity, specificity, positive likelihood ratio, negative likelihood ratio and diagnostic odds ratio were: 0.88 (95% CI: 0.85 ~ 0.91), 0.92 (95%CI: 0.90 ~ 0.94), 11.4 (95%CI: 9.1 ~14.3), 0.13 (95%CI: 0.10 ~ 0.17), and 88 (95%CI: 61 ~ 128), respectively (**Figures 3A, B**). Publication bias was not observed among these models in the training set (**Figure 3C**), and the prevalence of IP in the included studies was about 32%. Based on this prevalence, we established a prior probability of 32%. If the machine learning suggested IP, then the posterior probability of diagnosis of IP in positive patients was 84% and that of diagnosis of IP in negative patients was 6% (**Figure 3D**).

##### 3.4.1.2 Validation set

**Figure 4:**
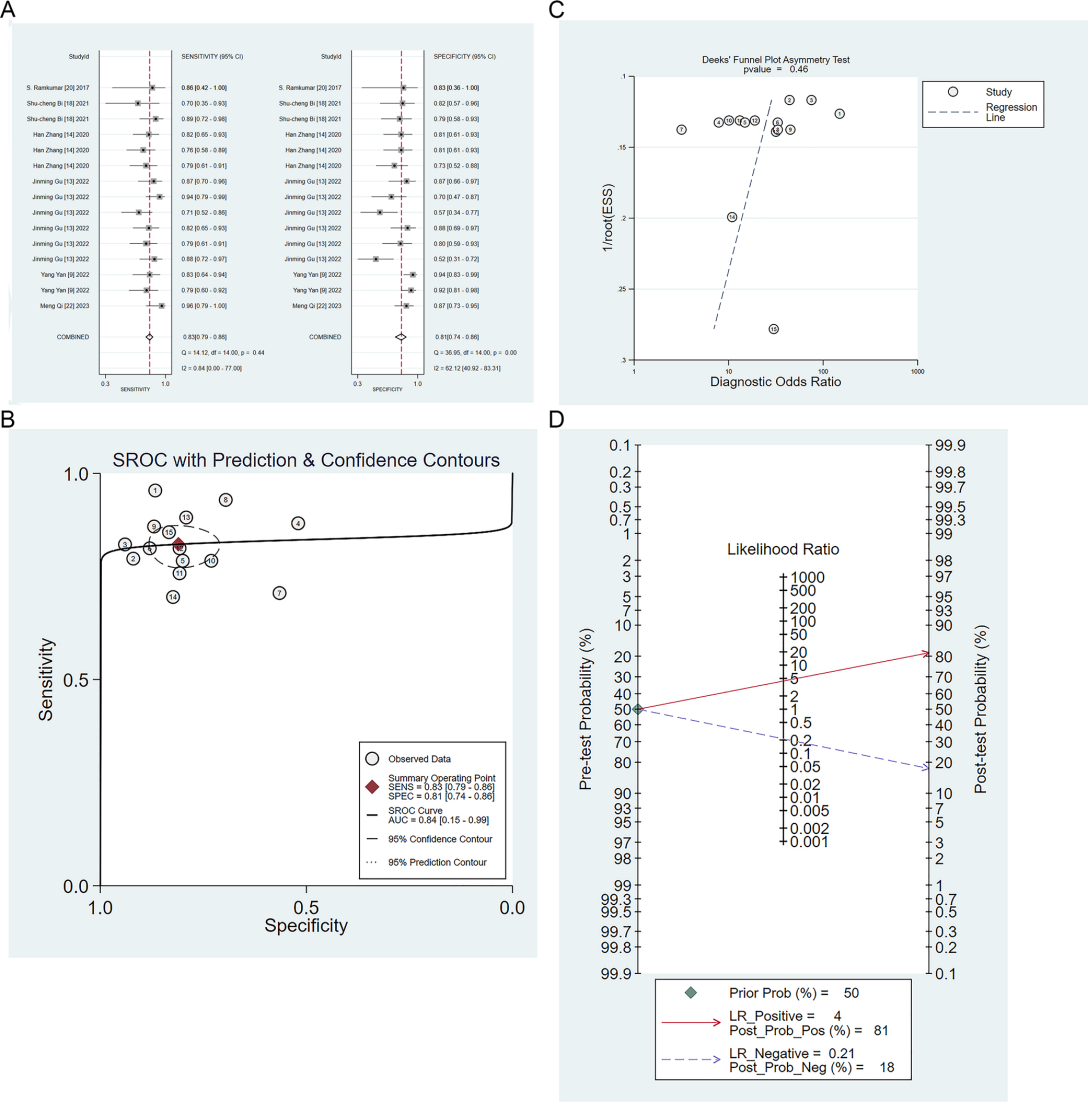
**(A)** Forest map of machine learning for identifying IP and nasal malignancies in the validation set. **(B)** SROC of machine learning for identifying IP and nasal malignancy in the validation set. **(C)** Funnel plot of machine learning for identifying IP and nasal malignancies in the validation set. **(D)** Nomogram of machine learning for identifying IP and nasal malignancies in the validation set.

Six studies validated the performance of machine learning to differentiate IP from malignant tumors (9, 13, 14, 18, 23, 24). The sensitivity, specificity, positive likelihood ratio, negative likelihood ratio, and diagnostic odds ratio of machine learning to differentiate IP from malignant tumors were 0.83 (95% CI: 0.79 ~ 0.86), 0.81 (95% CI: 0.74 ~ 0.86), 4.4 (95% CI: 3.2 ~6.0), 0.21 (95% CI: 0.17 ~ 0.27), and 21 (95% CI: 13 ~ 33), respectively (**Figures 4A, B**). Publication bias was identified among these models in the validation set (**Figure 4C**), and the prevalence of IP in the included studies was about 50%. Based on this prevalence, we established a prior probability of 50%. If the machine learning suggested IP, then the posterior probability of diagnosis of IP in positive patients was 81% and that of diagnosis of IP in negative patients was 18% (**Figure 4D**).

**Figure 5:**
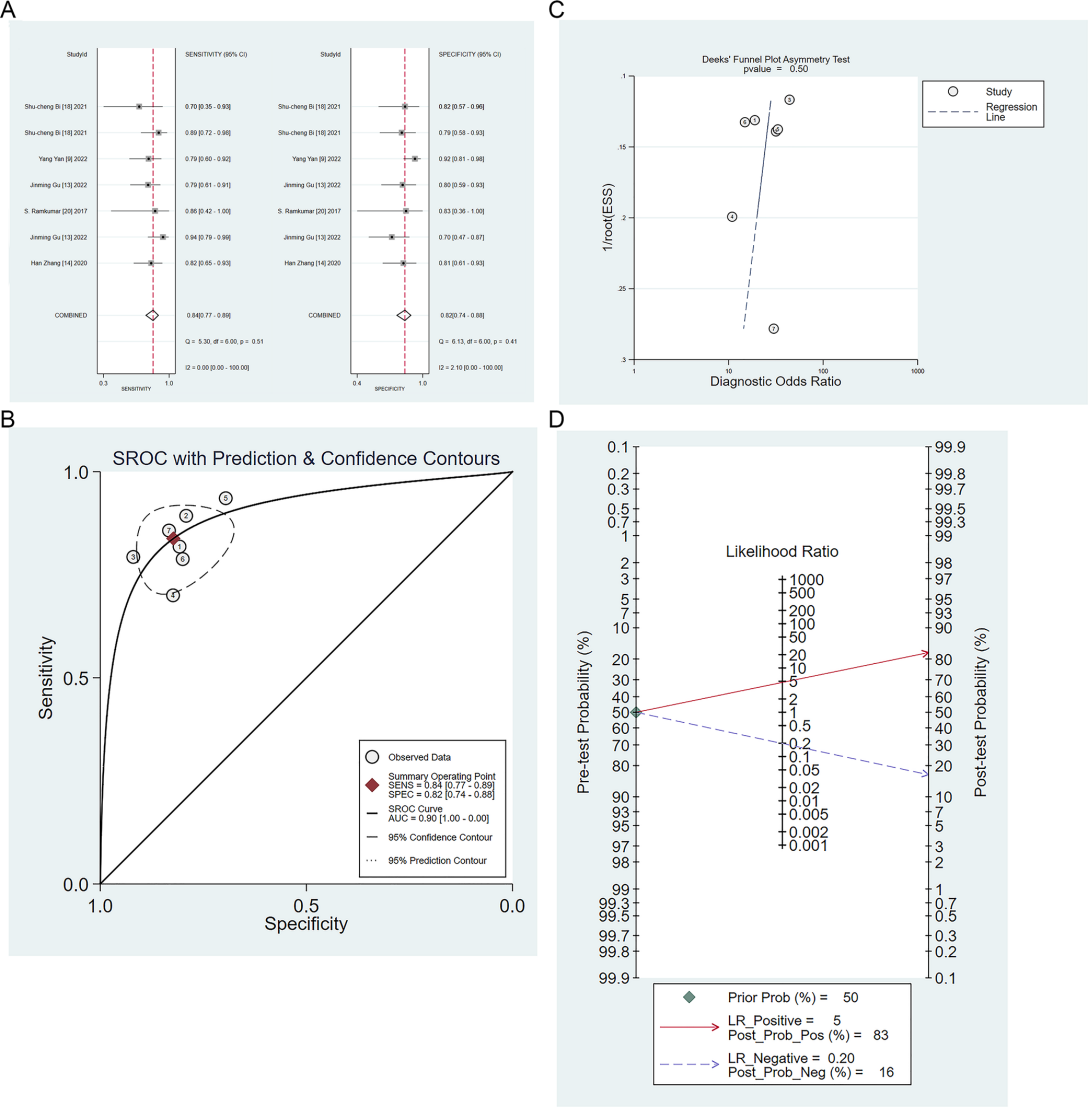
**(A)** Forest map of radiomics-based models for identifying IP and nasal malignancies in the validation set. **(B)** SROC of radiomics-based models for identifying IP and nasal malignancy in the validation set. **(C)** Funnel plot of radiomics-based models for identifying IP and nasal malignancies in the validation set. **(D)** Nomogram of radiomics-based models for identifying IP and nasal malignancies in the validation set.

Five studies validated the performance of radiomics-based models for the differential diagnosis of IP and malignant tumors (9, 13, 14, 18, 23). The sensitivity, specificity, positive likelihood ratio, negative likelihood ratio, and diagnostic odds ratio of radiomics-based models for identifying IP and malignant tumors were 0.84 (95% CI: 0.77 ~ 0.89), 0.82 (95%CI: 0.74 ~ 0.88), 4.7 (95%CI: 3.3 ~6.9), 0.20 (95%CI: 0.14 ~ 0.29), 24 (95%CI: 13 ~ 43), respectively (**Figures 5A, B**). There was no publication bias among these models in the validation set (**Figure 5C**), and the prevalence of IP in the included studies was about 50%. Based on this prevalence, we established a prior probability of 50%. If the machine learning suggested IP, then the posterior probability of diagnosis of IP in positive patients was 83% and that of diagnosis of IP in negative patients was 16% (**Figure 5D**).

Four studies (9, 13, 14, 24) validated the performance of machine learning models based on radiomic and clinical features to differentiate IP from malignant tumors. The analysis of these models revealed that the sensitivity, specificity, positive likelihood ratio, negative likelihood ratio, and diagnostic odds ratio were 0.85 (95% CI: 0.78 ~ 0.90), 0.87 (95%CI: 0.80 ~ 0.92), 6.5 (95%CI: 4.0 ~10.5), 0.18 (95%CI: 0.12 ~ 0.26), and 37 (95%CI: 18 ~ 77), respectively (**Supplementary Figure S3A, B**). There was no publication bias among these models in the validation set (**Supplementary Figure S3C**), and the prevalence of IP in the included studies was about 47%. Based on this prevalence, we established a prior probability of 47%. If the machine learning suggested IP, then the posterior probability of diagnosis of IP in positive patients was 85% and that of diagnosis of IP in negative patients was 13% (**Supplementary Figure S3D**).

#### 3.4.2 IP vs. benign lesions

Benign lesions, including sinusitis and nasal polyps, share similar clinical symptoms with IP and thus require distinction. There were three studies on the differential diagnosis of IP and nasal polyps (11, 16, 17), with the sensitivity and specificity ranges of (0.7140~0.9060) and (0.8160~0.8970), respectively. In one study, the sensitivity and specificity of differential diagnosis of IP and sinusitis (12) ranged from 0.9212 to 0.9548 and 0.8899 to 0.9097, respectively.

### 3.5 Clinical features that play an important role in the machine learning process

Three studies (7, 14, 25) constructed machine learning models based on clinical features for the differential diagnosis of IP and malignant tumors. The sensitivity and specificity were (0.5858~0.8790) and (0.4660~0.8001), respectively. Therefore, the role of clinical features should be considered in the development of machine learning models in the future.

## 4 Discussion

It has been found that the differential diagnosis of IP and malignancy relying on radiomics alone does not yield the best results. Therefore, we recommend integrating clinical features to improve the effectiveness of machine learning in IP differential diagnosis.

Imaging methods (such as CT or MRI) the first choice for the IP diagnosis (26, 27). In particular, there have been limited attempts to explore the diagnostic accuracy of CT or MRI for IP. Li Z et al. (28) investigated the diagnostic performance of dynamic contrast-enhanced MRI-derived parameters in distinguishing IP from squamous cell carcinoma. The AUC of the combination of the volume of extravascular extracellular space and rate constant was 0.831, with a specificity of 83% and sensitivity of 76.5%. However, effective preoperative diagnosis of IP remained a challenge. Radiomics methods can help determine the extent of tumors. This study evaluated the diagnostic performance of radiomics-based models in distinguishing IP from malignant tumors and benign lesions, which can not only improve the accuracy of diagnosis but also help increase the probability of complete surgical resection of tumors (20). Our analysis showed that the radiomics-based models showed high sensitivity [0.84 (95%CI: 0.77-0.89)] and specificity [0.82 (95% CI: 0.74 ~ 0.88)] in the validation set. This highlighted the importance of expanding the application of radiomics in the differential diagnosis of IP.

While our study focused on radiomics, we could not ignore the differences in clinical variables between IP and malignancy, such as age, smoking, and alcohol dependence. For instance, Hong SL et al. (29) discovered a correlation between smoking and malignant transformation in IP patients.

We found that there may be a high degree of bias in the modeling process. First, the number of cases in the modeling process should exceed 20, however, only a few included studies meet this condition, which may lead to overfitting of the constructed models. Second, model validation is required. At present, common clinical validation methods can be divided into internal validation and external validation. Internal verification is established according to the specific distribution trend of the data, mainly using random sampling. Random sampling can’t change the data distribution to some extent; Therefore, it does not explain the universality of this model. Only one study used prospective external validation and the remaining studies used random sampling. Third, there was a lack of consideration of overfitting in the included literature. Fourth, variable screening methods were different. Fifth, the selection of the model was predominantly based on logistic regression. While logistic regression is valuable in clinical applications with strong interpretability, there are limitations related to its application in radiomics.

Furthermore, another aspect that should be considered in the implementation of radiomics is the ignorance of variations in equipment, both across different manufacturers and different parameters within the same manufacturer. This oversight failed to address the potential effects of equipment over-configuration. This limitation highlights that radiomics is still in the theoretical stage and it is difficult to implement. It is important to formulate standardized research guidelines for future research and promote the development of radiomics according to the research guidelines.

For the first time, we explored the use of AI to distinguish IP from benign lesions and malignant tumors, and confirmed the feasibility of this approach. However, there are some limitations. First, due to the limited number of included studies, we did not specifically discuss different machine learning types under the same modeling variables. The predictive performance of various machine learning methods varies greatly. Future research should consider comparing the diagnostic performance of different machine learning methods for IP and developing intelligent assessment tools using the best performing machine learning methods. Secondly, the validation set is mainly generated by random sampling, and an independent external validation data set is lacking. Therefore, the results of the validation set need to be further verified.

## 5 Conclusions

Artificial intelligence methods can greatly improve the diagnosis of IP and reduce the misdiagnosis rate, thereby providing favorable support for clinical work, such as the formulation of surgical plans, frequency of postoperative re-examination, and the assessment of prognosis. Furthermore, artificial intelligence methods can be used to accurately identify the extent of a tumor, thus greatly increasing the probability of complete surgical resection and reducing the risk of recurrence. However, due to the small number of included studies, more prospective studies are needed to validate and develop universal radiomics-based diagnostic tools.

## Data Availability

The original contributions presented in the study are included in the article/**Supplementary Material**. Further inquiries can be directed to the corresponding authors.

## Supplementary material

Supplementary Table 1 Literature search strategy.
