# Supplementary material for: Identifying sinonasal inverted papilloma by machine learning: a systematic review and meta-analysis
Source: Front Oncol. 2025 Aug 26;15:1628999. doi: 10.3389/fonc.2025.1628999 (PMC12417127; doi:10.3389/fonc.2025.1628999)
Supplement: Supplementary Table 1 — Literature search strategy. [file DataSheet1.docx]

# Table S1 Literature search strategy

**1.Pubmed**

| Search number | Query |
| --- | --- |
| #1 | "Nose Neoplasms"[Mesh] |
| #2 | ((((((((((((((((((((((((((((Nose Neoplasms[Title/Abstract]) OR (Nose Neoplasm[Title/Abstract])) OR (Nasal Neoplasms[Title/Abstract])) OR (Nasal Neoplasm[Title/Abstract])) OR (Cancer of Nose[Title/Abstract])) OR (Nose Cancers[Title/Abstract])) OR (Nose Cancer[Title/Abstract])) OR (Nasal Cancer[Title/Abstract])) OR (Nasal Cancers[Title/Abstract])) OR (Cancer of the Nose[Title/Abstract])) OR (Sinonasal Tumors[Title/Abstract])) OR (Sinonasal Tumor[Title/Abstract])) OR (Inverted Papilloma[Title/Abstract])) OR (Inverted Papillomas[Title/Abstract])) OR (cancer of the nose[Title/Abstract])) OR (malignant nasal tumor[Title/Abstract])) OR (malignant tumor of the nose[Title/Abstract])) OR (nasal[Title/Abstract] AND paranasal cancer[Title/Abstract])) OR (nasal[Title/Abstract] AND paranasal sinus cancer[Title/Abstract])) OR (nasal[Title/Abstract] AND paranasal sinuses cancer[Title/Abstract])) OR (nasal carcinogenesis[Title/Abstract])) OR (nasal malignancies[Title/Abstract])) OR (nasal malignancy[Title/Abstract])) OR (sino-nasal cancer[Title/Abstract])) OR (sino-nasal malignancy[Title/Abstract])) OR (sinonasal cancer[Title/Abstract])) OR (sinonasal malignancy[Title/Abstract])) OR (sinonasal malignant neoplasm[Title/Abstract])) OR (sinonasal malignant tumor[Title/Abstract]) |
| #3 | ("Nose Neoplasms"[Mesh]) OR (((((((((((((((((((((((((((((Nose Neoplasms[Title/Abstract]) OR (Nose Neoplasm[Title/Abstract])) OR (Nasal Neoplasms[Title/Abstract])) OR (Nasal Neoplasm[Title/Abstract])) OR (Cancer of Nose[Title/Abstract])) OR (Nose Cancers[Title/Abstract])) OR (Nose Cancer[Title/Abstract])) OR (Nasal Cancer[Title/Abstract])) OR (Nasal Cancers[Title/Abstract])) OR (Cancer of the Nose[Title/Abstract])) OR (Sinonasal Tumors[Title/Abstract])) OR (Sinonasal Tumor[Title/Abstract])) OR (Inverted Papilloma[Title/Abstract])) OR (Inverted Papillomas[Title/Abstract])) OR (cancer of the nose[Title/Abstract])) OR (malignant nasal tumor[Title/Abstract])) OR (malignant tumor of the nose[Title/Abstract])) OR (nasal[Title/Abstract] AND paranasal cancer[Title/Abstract])) OR (nasal[Title/Abstract] AND paranasal sinus cancer[Title/Abstract])) OR (nasal[Title/Abstract] AND paranasal sinuses cancer[Title/Abstract])) OR (nasal carcinogenesis[Title/Abstract])) OR (nasal malignancies[Title/Abstract])) OR (nasal malignancy[Title/Abstract])) OR (sino-nasal cancer[Title/Abstract])) OR (sino-nasal malignancy[Title/Abstract])) OR (sinonasal cancer[Title/Abstract])) OR (sinonasal malignancy[Title/Abstract])) OR (sinonasal malignant neoplasm[Title/Abstract])) OR (sinonasal malignant tumor[Title/Abstract])) |
| #4 | "Machine Learning"[Mesh] |
| #5 | (((((((((((((((((((((((Transfer Learning[Title/Abstract]) OR (Deep learning[Title/Abstract])) OR (Ensemble Learning[Title/Abstract])) OR (artificial intelligence[Title/Abstract])) OR (Prediction model[Title/Abstract])) OR (random forest[Title/Abstract])) OR (neural network[Title/Abstract])) OR (neural networks[Title/Abstract])) OR (CNN[Title/Abstract])) OR (Support vector machine[Title/Abstract])) OR (SVM[Title/Abstract])) OR (Gradient Boosting Machine[Title/Abstract])) OR (GBM[Title/Abstract])) OR (Nomogram[Title/Abstract])) OR (XGBoost[Title/Abstract])) OR (Adaboost[Title/Abstract])) OR (Decision tree[Title/Abstract])) OR (ResNet-50[Title/Abstract])) OR (ResNet[Title/Abstract])) OR (Radiomics[Title/Abstract])) OR (Radiomic[Title/Abstract])) OR (Naive Bayesian[Title/Abstract])) OR (Multilayer perceptron[Title/Abstract])) OR (Bayesian network[Title/Abstract]) |
| #6 | ("Machine Learning"[Mesh]) OR ((((((((((((((((((((((((Transfer Learning[Title/Abstract]) OR (Deep learning[Title/Abstract])) OR (Ensemble Learning[Title/Abstract])) OR (artificial intelligence[Title/Abstract])) OR (Prediction model[Title/Abstract])) OR (random forest[Title/Abstract])) OR (neural network[Title/Abstract])) OR (neural networks[Title/Abstract])) OR (CNN[Title/Abstract])) OR (Support vector machine[Title/Abstract])) OR (SVM[Title/Abstract])) OR (Gradient Boosting Machine[Title/Abstract])) OR (GBM[Title/Abstract])) OR (Nomogram[Title/Abstract])) OR (XGBoost[Title/Abstract])) OR (Adaboost[Title/Abstract])) OR (Decision tree[Title/Abstract])) OR (ResNet-50[Title/Abstract])) OR (ResNet[Title/Abstract])) OR (Radiomics[Title/Abstract])) OR (Radiomic[Title/Abstract])) OR (Naive Bayesian[Title/Abstract])) OR (Multilayer perceptron[Title/Abstract])) OR (Bayesian network[Title/Abstract])) |
| #7 | (("Nose Neoplasms"[Mesh]) OR (((((((((((((((((((((((((((((Nose Neoplasms[Title/Abstract]) OR (Nose Neoplasm[Title/Abstract])) OR (Nasal Neoplasms[Title/Abstract])) OR (Nasal Neoplasm[Title/Abstract])) OR (Cancer of Nose[Title/Abstract])) OR (Nose Cancers[Title/Abstract])) OR (Nose Cancer[Title/Abstract])) OR (Nasal Cancer[Title/Abstract])) OR (Nasal Cancers[Title/Abstract])) OR (Cancer of the Nose[Title/Abstract])) OR (Sinonasal Tumors[Title/Abstract])) OR (Sinonasal Tumor[Title/Abstract])) OR (Inverted Papilloma[Title/Abstract])) OR (Inverted Papillomas[Title/Abstract])) OR (cancer of the nose[Title/Abstract])) OR (malignant nasal tumor[Title/Abstract])) OR (malignant tumor of the nose[Title/Abstract])) OR (nasal[Title/Abstract] AND paranasal cancer[Title/Abstract])) OR (nasal[Title/Abstract] AND paranasal sinus cancer[Title/Abstract])) OR (nasal[Title/Abstract] AND paranasal sinuses cancer[Title/Abstract])) OR (nasal carcinogenesis[Title/Abstract])) OR (nasal malignancies[Title/Abstract])) OR (nasal malignancy[Title/Abstract])) OR (sino-nasal cancer[Title/Abstract])) OR (sino-nasal malignancy[Title/Abstract])) OR (sinonasal cancer[Title/Abstract])) OR (sinonasal malignancy[Title/Abstract])) OR (sinonasal malignant neoplasm[Title/Abstract])) OR (sinonasal malignant tumor[Title/Abstract]))) AND (("Machine Learning"[Mesh]) OR ((((((((((((((((((((((((Transfer Learning[Title/Abstract]) OR (Deep learning[Title/Abstract])) OR (Ensemble Learning[Title/Abstract])) OR (artificial intelligence[Title/Abstract])) OR (Prediction model[Title/Abstract])) OR (random forest[Title/Abstract])) OR (neural network[Title/Abstract])) OR (neural networks[Title/Abstract])) OR (CNN[Title/Abstract])) OR (Support vector machine[Title/Abstract])) OR (SVM[Title/Abstract])) OR (Gradient Boosting Machine[Title/Abstract])) OR (GBM[Title/Abstract])) OR (Nomogram[Title/Abstract])) OR (XGBoost[Title/Abstract])) OR (Adaboost[Title/Abstract])) OR (Decision tree[Title/Abstract])) OR (ResNet-50[Title/Abstract])) OR (ResNet[Title/Abstract])) OR (Radiomics[Title/Abstract])) OR (Radiomic[Title/Abstract])) OR (Naive Bayesian[Title/Abstract])) OR (Multilayer perceptron[Title/Abstract])) OR (Bayesian network[Title/Abstract]))) |

**2.Cochrane**

| Search number | Query |
| --- | --- |
| #1 | MeSH descriptor: [Nose Neoplasms] explode all trees |
| #2 | (Nose Neoplasms):ti,ab,kw OR (Nose Neoplasm):ti,ab,kw OR (Nasal Neoplasms):ti,ab,kw OR (Nasal Neoplasm):ti,ab,kw OR (Cancer of Nose):ti,ab,kw |
| #3 | (Nose Cancers):ti,ab,kw OR (Nose Cancer):ti,ab,kw OR (Nasal Cancer):ti,ab,kw OR (Nasal Cancers):ti,ab,kw OR (Cancer of the Nose):ti,ab,kw |
| #4 | (Sinonasal Tumors):ti,ab,kw OR (cancer of the nose):ti,ab,kw OR (Sinonasal Tumor):ti,ab,kw OR (Inverted Papilloma):ti,ab,kw OR (Inverted Papillomas):ti,ab,kw |
| #5 | (malignant nasal tumor):ti,ab,kw OR (malignant tumor of the nose):ti,ab,kw OR (nasal and paranasal cancer):ti,ab,kw OR (nasal and paranasal sinus cancer):ti,ab,kw OR (nasal and paranasal sinuses cancer):ti,ab,kw |
| #6 | (nasal carcinogenesis):ti,ab,kw OR (nasal malignancies):ti,ab,kw OR (nasal malignancy):ti,ab,kw OR (sino-nasal cancer):ti,ab,kw OR (sino-nasal malignancy):ti,ab,kw |
| #7 | (sinonasal cancer):ti,ab,kw OR (sinonasal malignancy):ti,ab,kw OR (sinonasal malignant neoplasm):ti,ab,kw OR (sinonasal malignant tumor):ti,ab,kw |
| #8 | #1 or #2 or #3 or #4 or #5 or #6 or #7 |
| #9 | MeSH descriptor: [Machine Learning] explode all trees |
| #10 | (machine learning):ti,ab,kw OR (Transfer Learning):ti,ab,kw OR (Deep learning):ti,ab,kw OR (Ensemble Learning):ti,ab,kw OR (artificial intelligence):ti,ab,kw |
| #11 | (Prediction model):ti,ab,kw OR (random forest):ti,ab,kw OR (neural network):ti,ab,kw OR (neural networks):ti,ab,kw OR (CNN):ti,ab,kw |
| #12 | (Support vector machine):ti,ab,kw OR (SVM):ti,ab,kw OR (Gradient Boosting Machine):ti,ab,kw OR (GBM):ti,ab,kw OR (Nomogram):ti,ab,kw |
| #13 | (XGBoost):ti,ab,kw OR (Adaboost):ti,ab,kw OR (Decision tree):ti,ab,kw OR (ResNet-50):ti,ab,kw OR (ResNet):ti,ab,kw |
| #14 | (Radiomics):ti,ab,kw OR (Radiomic):ti,ab,kw OR (Naive Bayesian):ti,ab,kw OR (Multilayer perceptron):ti,ab,kw OR (Bayesian network):ti,ab,kw |
| #15 | #9 or #10 or #11 or #12 or #13 or #14 |
| #16 | #8 and #15 |

**3.Embase**

| Search number | Query |
| --- | --- |
| #1 | 'machine learning'/exp |
| #2 | 'machine learning':ab,ti OR 'transfer learning':ab,ti OR 'deep learning':ab,ti OR 'ensemble learning':ab,ti OR 'artificial intelligence':ab,ti OR 'prediction model':ab,ti OR 'random forest':ab,ti OR 'neural network':ab,ti OR 'neural networks':ab,ti OR cnn:ab,ti OR 'support vector machine':ab,ti OR svm:ab,ti OR 'gradient boosting machine':ab,ti OR gbm:ab,ti OR nomogram:ab,ti OR xgboost:ab,ti OR adaboost:ab,ti OR 'decision tree':ab,ti OR 'resnet 50':ab,ti OR resnet:ab,ti OR radiomics:ab,ti OR radiomic:ab,ti OR 'naive bayesian':ab,ti OR 'multilayer perceptron':ab,ti OR 'bayesian network':ab,ti |
| #3 | #1 OR #2 |
| #4 | 'nose tumor'/exp |
| #5 | 'nose neoplasms':ab,ti OR 'nose neoplasm':ab,ti OR 'nasal neoplasms':ab,ti OR 'nasal neoplasm':ab,ti OR 'cancer of nose':ab,ti OR 'nose cancers':ab,ti OR 'nose cancer':ab,ti OR 'nasal cancer':ab,ti OR 'nasal cancers':ab,ti OR 'sinonasal tumors':ab,ti OR 'sinonasal tumor':ab,ti OR 'inverted papilloma':ab,ti OR 'inverted papillomas':ab,ti OR 'cancer of the nose':ab,ti OR 'malignant nasal tumor':ab,ti OR 'malignant tumor of the nose':ab,ti OR (nasal:ab,ti AND 'paranasal cancer':ab,ti) OR (nasal:ab,ti AND 'paranasal sinus cancer':ab,ti) OR (nasal:ab,ti AND 'paranasal sinuses cancer':ab,ti) OR 'nasal carcinogenesis':ab,ti OR 'nasal malignancies':ab,ti OR 'nasal malignancy':ab,ti OR 'sino-nasal cancer':ab,ti OR 'sino-nasal malignancy':ab,ti OR 'sinonasal cancer':ab,ti OR 'sinonasal malignancy':ab,ti OR 'sinonasal malignant neoplasm':ab,ti OR 'sinonasal malignant tumor':ab,ti |
| #6 | #4 OR #5 |
| #7 | #3 AND #6 |

**4.Web of science**

| Search number | Query |
| --- | --- |
| #1 | Nose Neoplasms (Topic) OR Nose Neoplasm (Topic) OR Nasal Neoplasms (Topic) OR Nasal Neoplasm (Topic) OR Cancer of Nose (Topic) OR Nose Cancers (Topic) OR Nose Cancer (Topic) OR Nasal Cancer (Topic) OR Nasal Cancers (Topic) OR Cancer of the Nose (Topic) OR Sinonasal Tumors (Topic) OR Sinonasal Tumor (Topic) OR Inverted Papilloma (Topic) OR Inverted Papillomas (Topic) OR cancer of the nose (Topic) OR malignant nasal tumor (Topic) OR malignant tumor of the nose (Topic) OR nasal and paranasal cancer (Topic) OR nasal and paranasal sinus cancer (Topic) OR nasal and paranasal sinuses cancer (Topic) OR nasal carcinogenesis (Topic) OR nasal malignancies (Topic) OR nasal malignancy (Topic) OR sino-nasal cancer (Topic) OR sino-nasal malignancy (Topic) OR sinonasal cancer (Topic) OR sinonasal malignancy (Topic) OR sinonasal malignant neoplasm (Topic) OR sinonasal malignant tumor (Topic) |
| #2 | machine learning (Topic) OR Transfer Learning (Topic) OR Deep learning (Topic) OR Ensemble Learning (Topic) OR artificial intelligence (Topic) OR Prediction model (Topic) OR random forest (Topic) OR neural network (Topic) OR neural networks (Topic) OR CNN (Topic) OR Support vector machine (Topic) OR SVM (Topic) OR Gradient Boosting Machine (Topic) OR GBM (Topic) OR Nomogram (Topic) OR XGBoost (Topic) OR Adaboost (Topic) OR Decision tree (Topic) OR ResNet-50 (Topic) OR ResNet (Topic) OR Radiomics (Topic) OR Radiomic (Topic) OR Naive Bayesian (Topic) OR Multilayer perceptron (Topic) OR Bayesian network (Topic) |
| #3 | #2 AND #1 |


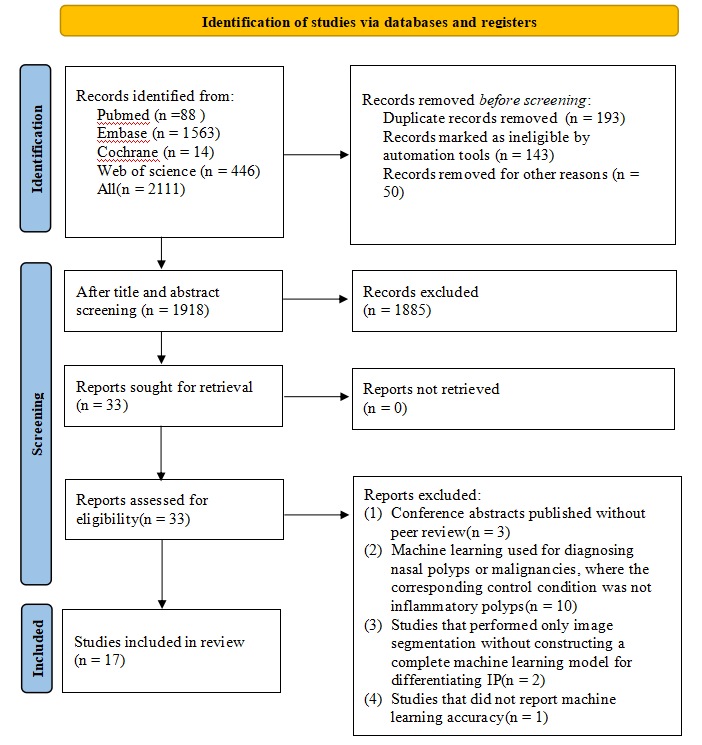


**Figure S1** Flowchart of study selection.


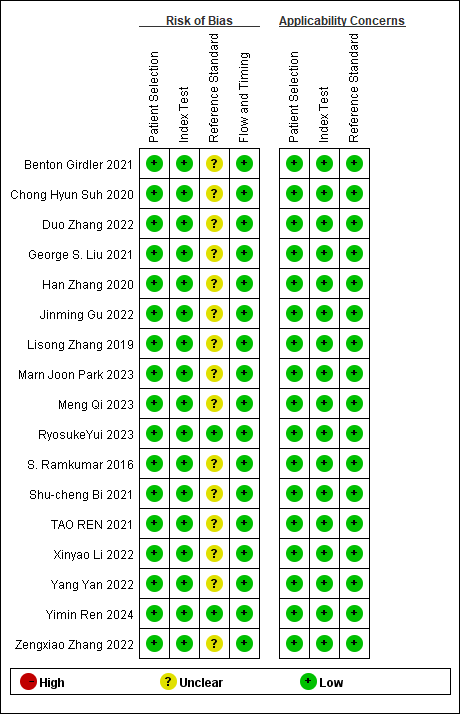


**Figure S2** Results of risk of bias assessment in the included studies.


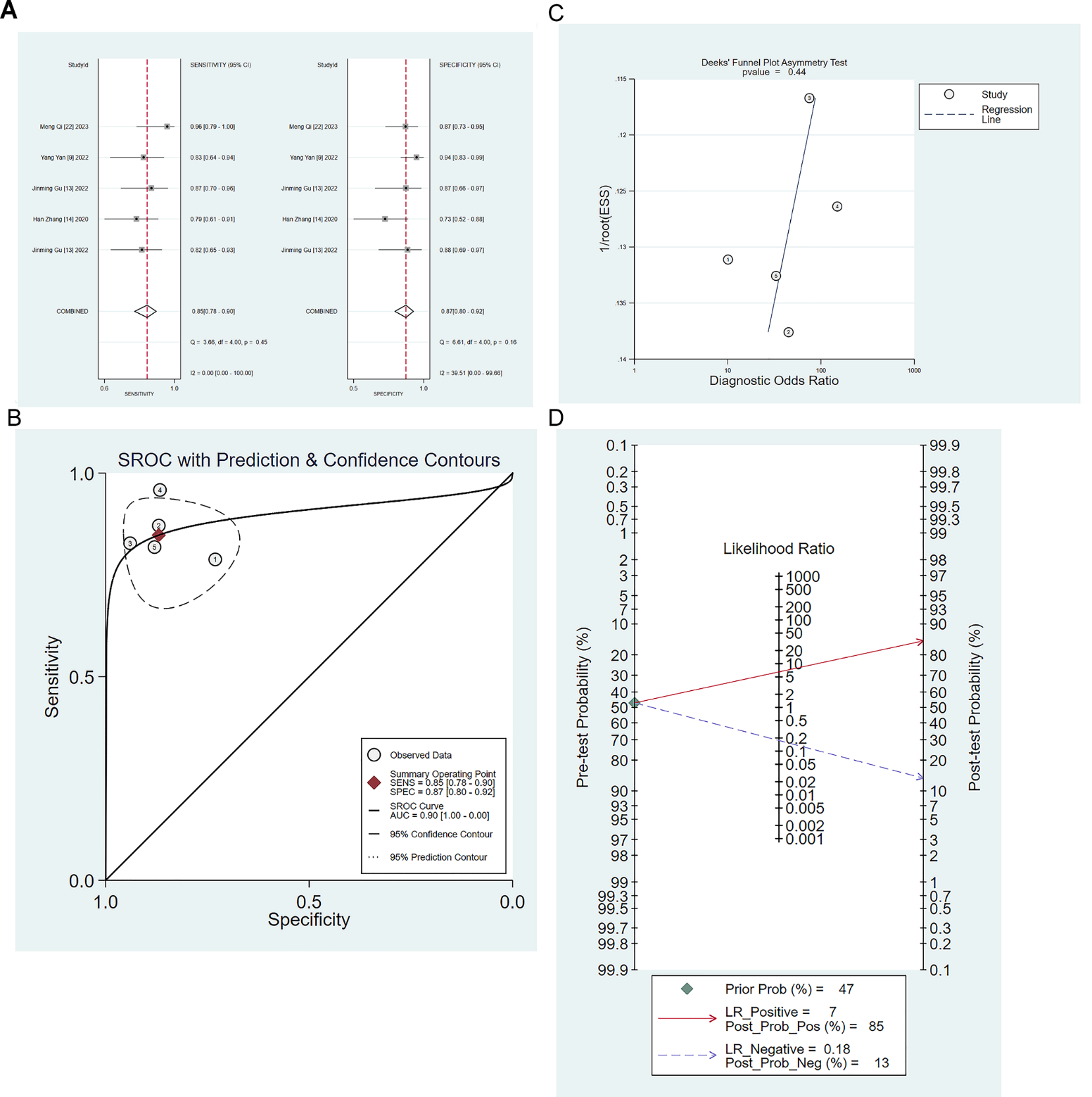


**Figure S3** **A:** Forest map of machine learning models based on radiomic and clinical features for identifying IP and nasal malignancies in the validation set. **B:** SROC of machine learning models based on radiomic and clinical features for identifying IP and nasal malignancy in the validation set. **C:** Funnel plot of machine learning models based on radiomic and clinical features for identifying IP and nasal malignancies in the validation set. **D:** Nomogram of machine learning models based on radiomic and clinical features for identifying IP and nasal malignancies in the validation set.
